# Plumbagin alleviates obesity‐related asthma: Targeting inflammation, oxidative stress, and the AMPK pathway

**DOI:** 10.1002/iid3.1025

**Published:** 2023-09-27

**Authors:** Lijie Zhang, Dongxue Liang, Linlin Liu, Lihua Liu

**Affiliations:** ^1^ Second Ward of Respiratory Department The First Affiliated Hospital of Jinzhou Medical University Jinzhou Liaoning People's Republic of China; ^2^ Ward of Respiratory and Critical Care Department The First Affiliated Hospital of Jinzhou Medical University Jinzhou Liaoning People's Republic of China

**Keywords:** AMPK, inflammation, obesity‐related asthma, oxidative stress, plumbagin

## Abstract

**Background:**

Obesity‐related asthma, a specific type of asthma, tends to have more severe symptoms and more frequent exacerbations, and it is insensitive to standard medications. Plumbagin (PLB) has many positive effects on human health. However, it remains unclear whether PLB protects against obesity‐related asthma. The study investigated the effect of PLB on obesity‐related asthma.

**Methods:**

Four‐week‐old male C57BL6/J mice were fed either standard‐chow diet or high‐fat diet (HFD). The mice were sensitized to 100 μg ovalbumin (OVA) once a week and intraperitoneally injected with 1 mg/kg PLB once daily from Week 10 to 11 and then challenged with 10 μg OVA twice a day on Week 12. The lung tissue and bronchoalveolar lavage fluid (BALF) were collected 48 h after the first OVA challenge.

**Results:**

HFD enhanced inflammatory cell infiltration within the airways and increased total inflammatory cell and eosinophil counts, levels of eosinophil‐related inflammatory cytokines, including interleukin‐4 (IL‐4), IL‐5, and eotaxin in BALF, and oxidative stress in the lung tissues of asthmatic mice. PLB reduced inflammatory cell infiltration in the airway walls, levels of eosinophil‐related inflammatory cytokines in BALF, and oxidative stress in lung tissues of obese asthmatic mice. In addition, PLB restored HFD‐induced decreases in adenosine monophosphate‐activated protein kinase (AMPK) phosphorylation.

**Conclusion:**

The study suggested that HFD exacerbated inflammation and oxidative stress, while PLB probably alleviated inflammation and oxidative stress and activated AMPK pathway to attenuate obesity‐associated asthma. Thus, PLB likely had the potential to treat obesity‐related asthma.

## Graphical abstract

High‐fat diet exacerbated inflammation and oxidative stress, while plumbagin probably alleviated inflammation and oxidative stress and activated AMPK pathway to prevent obesity‐associated asthma.

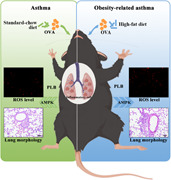

## 1 INTRODUCTION

Asthma (bronchial asthma) is one of the chronic airway inflammatory diseases that seriously threaten human health,^1^ which leads to airway hyperresponsiveness (AHR), reversible obstruction, as well as the recurrent episodes of wheezing, dyspnea, and coughing.^2^, ^3^ Those symptoms often occur at night and/or in the morning, and most of them are relieved spontaneously or by treatment. Patients with asthma often experience an exacerbation in their lives, which may be life‐threatening. Approximately 235–300 million people suffer from asthma worldwide and 180,000 deaths annually due to asthma.^4^ The World Health Organization predicted that the number of asthma sufferers would rise to 400 million by 2025.^5^ The prevalence of asthma imposes a huge burden on patients, their families, and society.

The occurrence of asthma is influenced by a variety of genetic factors and environmental factors.^6^ Among many factors, the influence of obesity on asthma has become the focus of attention. A clinical research showed that obesity increased asthma severity.^7^ Accumlating evidence has suggested that increased body mass index was linked to allergic and nonallergic asthma.^8^ There was disagreement about the regulatory mechanism of obesity‐related asthma. In obesity, adipose tissues produce proinflammatory cytokines such as interleukin‐6 (IL‐6) and tumor necrosis factor‐α (TNF‐α), and thus obesity is regarded as a chronic systemic inflammatory disease. Obesity aggravates airway inflammation^9^ and promotes eosinophilic inflammation in allergic asthma.^10^ Obese asthmatics have elevated levels of oxidative stress and systemic inflammation that influenced the severity of airway inflammation in asthmatics.^11^ Nevertheless, Sideleva et al.^12^ found that airway inflammation was reduced in obese asthmatic patients, and they considered that adipokines contributed to asthma by directly affecting the airway rather than by promoting inflammation. Due to standard drug tolerance, obese asthmatics are prone to frequent exacerbations and worse quality of life.^13^ Obesity‐related asthma becomes a difficult problem in the treatment of asthma, and thus new therapeutic drugs for obesity‐related asthma are urgently needed.

Plant‐derived natural products have been proven to be effective in treating various human diseases.^14^, ^15^ Plumbagin (PLB), isolated from the roots of *Plumbago zeylanica* L, is a natural vitamin K3 analogue.^16^ Accumulating evidence suggest that the elevated oxidative stress during asthma is linked to airway inflammation and AHR.^17^, ^18^ PLB has been reported to protect against multiple diseases, due to its anti‐inflammatory,^19^, ^20^ antioxidant,^21^ and antianaphylactic^22^ properties. The effect of PLB in obesity‐related asthma remains unknown. PLB promoted AMPK phosphorylation.^23^ Activated AMPK attenuated oxidative stress and further alleviated allergic asthma.^24^ Based on this evidence, we hypothesized that PLB might have the potential in treating asthma. To validate our hypothesis, the effect of PLB on inflammation and oxidative stress was investigated in one mouse model of obesity‐related asthma.

## 2 MATERIALS AND METHODS

### 2.1 Reagents

Ovalbumin (OVA, #A107820), PLB (#P424180), and xylene (#1330‐20‐7) were purchased from Aladdin. Wright‐Giemsa staining fluid (#D010) and superoxide dismutase (SOD) assay kit (#A001) were from Nanjing Jiancheng Bioengineering Institute. Methanol (#10014118) and absolute ethyl alcohol (#10009218) were purchased from Sinopharm Chemical Reagent Co. Ltd. Hematoxylin (#H8070) and mounting medium (S2100) were purchased from Solarbio. Eosin Y was purchased from Sangon Biotech Co. Ltd. The mouse tumor TNF‐α enzyme‐linked immunosorbent assay (ELISA) kit (#EK282), the mouse IL‐4 ELISA kit (#EK204), the mouse IL‐5 ELISA kit (#EK205), and the mouse eosinophil chemokine (Eotaxin) ELISA kit (#EK2130) were purchased from Multisciences (Lianke) Biotech. The BCA protein determination assay kit (#P0011), protein lysis buffer (#P0013), and goat anti‐rabbit IgG (#A0208) were purchased from Beyotime. The reactive oxygen species (ROS) detection kit (#BB‐470513) was purchased from BestBio. AMPK (#AF6423) and phspho‐AMPK (p‐AMPK, #AF3423) antibodies were purchased from Affinity. The β‐actin (#sc‐47778) antibody was purchased from Santa Cruz Biotechnology.

### 2.2 Animals

Four‐week‐old male C57BL6/J mice were purchased from Liaoning Changsheng Biotechnology. All animals were given free access to water and standard‐chow diet (SCD) and maintained in a temperature‐controlled (25 ± 2°C) and humidity‐controlled (40%–60%) environment with a 12‐h light/dark cycle. All animal experiments were performed in accordance with the the Guide for the Care and Use of Laboratory Animals and were approved by Jinzhou Medical University Laboratory Animal Ethics Committee.

### 2.3 Animal treatment and grouping

**Figure 1:**
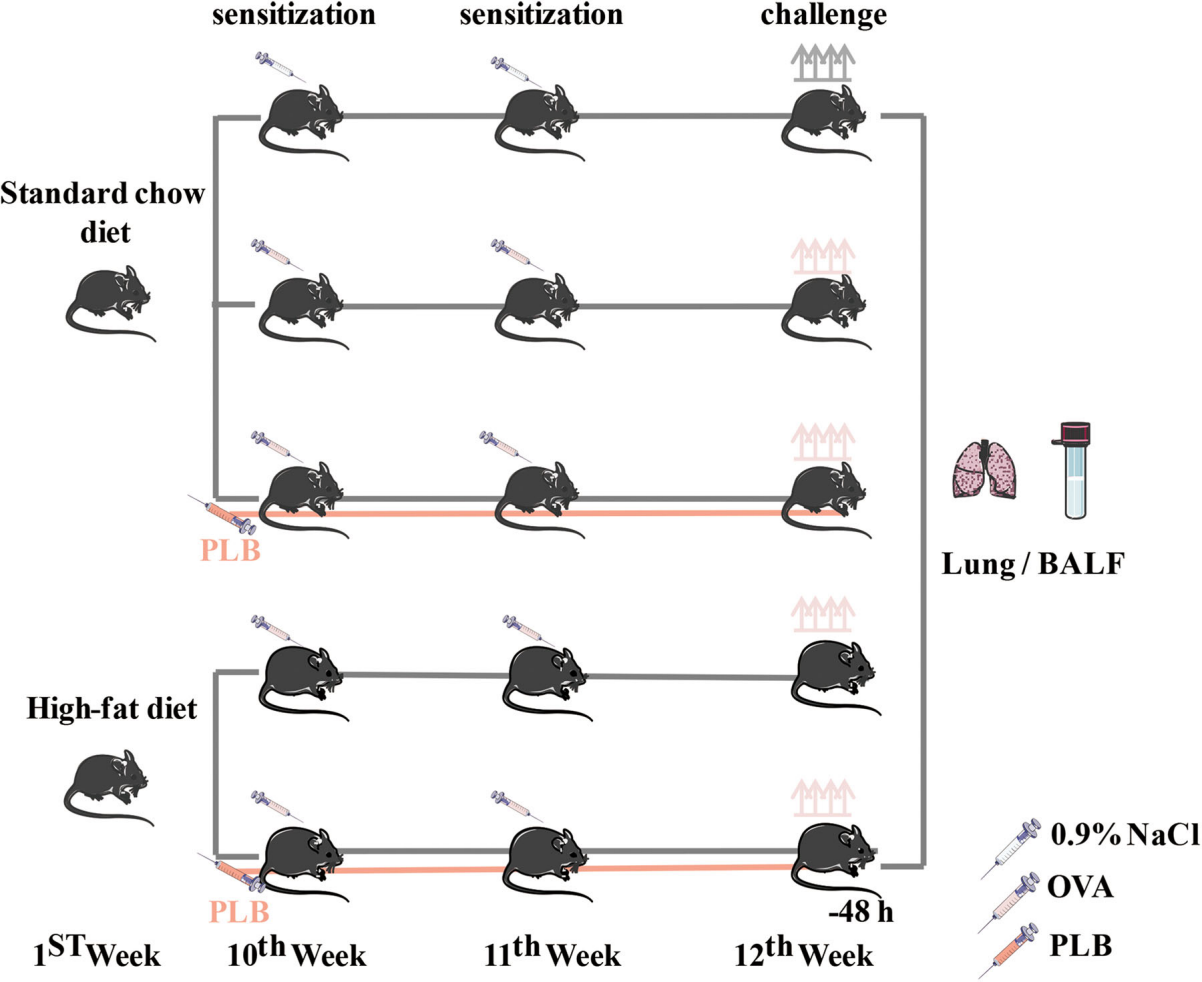
Schematic diagram of OVA sensitization and challenge and the time‐course of PLB treatment in mice fed SCD or HFD. BALF, bronchoalveolar lavage fluid; HFD, high‐fat diet; OVA, ovalbumin; PLB, plumbagin; SCD, standard‐chow diet.

After 1‐week acclimatization, a total of 80 mice were randomly divided into five groups: control group (*n* = 12), OVA group (*n* = 17), OVA + PLB group (*n* = 17), high‐fat diet (HFD) + OVA group (*n* = 17), and HFD + OVA + PLB group (*n* = 17). The mice were randomly divided into groups using a computer‐based random number generator (https://www.randomizer.org/). The number of animals required was inferred from the similar experiments previously conducted. The mice in the control, OVA, and OVA + PLB groups were fed SCD (10% fat) for 12 weeks and those in the HFD + OVA and HFD + OVA + PLB groups were fed HFD (55% fat) for 12 weeks to induce obesity. Induction of obesity was considered successful when the mice weighed 20% more than the body weight of the control mice. As shown in Figure 1, the mice in the OVA + PLB and HFD + OVA + PLB groups received 1 mg/kg PLB by intraperitoneal injection once a day for 14 consecutive days at Weeks 10 and 11. The order of PLB administration was randomized daily. The individual mouse was considered the experimental unit within the studies.

### 2.4 Sensitization and challenge

To develop an asthma model as previously described^25^, mice were subcutaneously injected 100 μg OVA on the first day of Weeks 10 and 11 for sensitization. The mice in the control group were given an equal volume of 0.9% NaCl. The OVA‐sensitized mice were challenged intranasally with OVA (10 μg/50 μL) twice a day on Day 1 and 2 of Week 12. Nonsensitized mice were identically challenged with 0.9% NaCl, as shown in Figure 1. The respiratory rate and the manifestations including fidgeting, sneezing, and wheezing were recorded daily. The animals without the above described manifestations were excluded. The number of mice used for experimental analysis was listed in Supporting Information: Table S1. Mice were euthanized at 48 h after the first OVA challenge. Bronchoalveolar lavage fluid (BALF) and the lung tissues were collected. The lung tissues were fixed or stored at −70°C. The epididymal fat mass was harvested and weighed. A total of 60 mice were used for experimental analyses with 12 mice per group. Histopathologic changes, ROS levels, SOD activities, and the levels of specific proteins in the lung tissues and the number of inflammatory cells and the level of cytokines in BALF were assessed. The investigator was blinded to the experimental grouping in the follow‐up test.

### 2.5 BALF collection and cell count

The fluid was centrifuged at 300*g* for 10 min, and the cell pellet was obtained and resuspended in 500 μL PBS. A blood cell counter was used to detect the total number of inflammatory cells in BALF. The Wright‐Giemsa staining kit was applied to calculate the number of eosinophils. Briefly, the resuspended BALF (10 μL) was transferred to a microscope slide, fixed with methanol for 15 min, stained with Giemsa A solution for 1 min and Giemsa B solution for 7 min, and decolorized with 80% ethanol. Finally, the slides were observed using the DP73 microscope photo system (Olympus). The cells with a purple nucleus, a pale pink cytoplasm, and orange‐red granules were regarded as eosinophils.

### 2.6 Histopathology analysis

The lung tissues were fixed with 4% paraformaldehyde, dehydrated with gradient ethanol, embedded in paraffin, and then sliced using a Leica microtome (RM2235) at 5 μm. The sections were baked at 60°C for 2 h, deparaffinized in xylene, and stained with hematoxylin and eosin (H&E) according to the manufacturer's instructions. The images were observed using a BX53 microscope (Olympus). Lung tissue inflammation was evaluated by couting the inflammatory cells, including granulocytes and mononuclear cells, within the airway wall area (WA) around the airways as previously described.^26^ Results were expressed as the number of inflammatory cells per square millimeter of WA.

### 2.7 ELISA

The levels of TNF‐α, IL‐4, IL‐5, and eotaxin in BALF were measured by the commercial ELISA kits referring to the manufacturer's instructions. Absorbance was detected using a ELX‐800 microplate reader (BioTek) at 450 and 570 nm.

### 2.8 Detection of SOD activities

The lung tissues were homogenized in ice bath and centrifuged at 2500 rpm for 10 min, and the supernatant was collected. The protein concentration was evaluated by performing BCA assays. The activity of SOD was detected using the SOD assay kit according to the manufacturer's instructions. The optical density was measured at 550 nm using the UV752N spectrophotometer (YOKE Instrument).

### 2.9 Detection of ROS levels

The levels of ROS were detected using the fluorescent probe BBoxiProbe® O13. The fresh lung tissues were embedded in OCT compound and sectioned with a cryostat microtome. The slices were dried and washed three times for 5 min with water. And then, the sections were incubated with BBoxiProbe® O13 for 30 min at 37°C, washed three times for 5 min in PBS, and subsequently mounted in mounting medium. The fluorescence images were captured using a BX53 microscope (Olympus).

### 2.10 Western blot analysis

The protein was extracted using lysis buffer. Briefly, protein extraction reagent was added to each samples, and the samples were immersed in ice‐bath for 5 min and then centrifuged at 10,000*g* for 5 min at 4°C. The supernatants were collected for western blot analysis. The protein concentration was quantified by performing BCA assays. Proteins were electrophoresed by sodium dodecyl sulfate‐polyacrylamide gel electrophoresis, and then electrotransferred onto the polyvinylidene difluoride (PVDF) membranes. The PVDF membranes were blocked with 5% skim milk at room temperature, followed by immunoblotting with the AMPK antibody or the p‐AMPK antibody overnight at 4°C. After incubation, the PVDF membranes were washed with TBST and subsequently conjugated with the secondary antibody. The protein bands were visualized using an enhanced chemiluminescence system.

### 2.11 Statistical analyses

Statistical analyses were performed using GraphPad Prism 8. The values were expressed as mean ± standard deviation (mean ± SD). Data were analyzed using unpaired *t* test and two‐way analysis of variance (ANOVA) followed by Tukey's test. The *p* <  .05 was suggested as statistically significant. All experiments were repeated six times (*N* = 6).

## 3 RESULTS

### 3.1 PLB reduced HFD‐induced obesity and epididymal fat mass in OVA‐challenged mice

**Figure 2:**
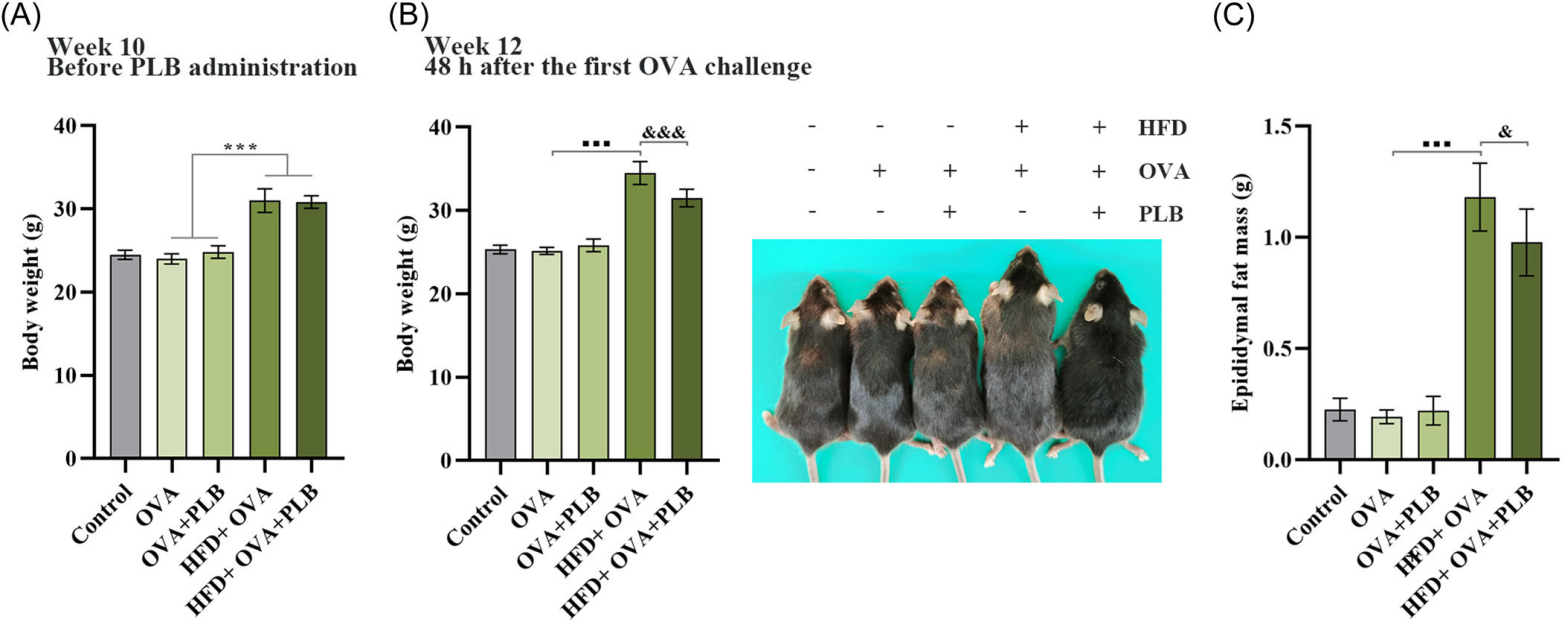
Effects of PLB on body weight and epididymal fat mass in nonobese and obese asthmatic mice. OVA‐challenged mice (fed SCD or HFD) were treated with PLB for 2 weeks. (A) Body weight before PLB treatment. ***p < .05. (B) Body weight at 48 h after first challenge of OVA and representative images of mice. (C) Epididymal fat mass at 48 h after first challenge of OVA. ^■■■^ *p* < .001 versus OVA group, ^&^ *p* < .05 and ^&&&^ *p* < .001 versus HFD + OVA group. Results were expressed as mean ± SD. *N* = 6. HFD, high‐fat diet; OVA, ovalbumin; PLB, plumbagin.

The 10‐week HFD feeding significantly increased the body weight of mice in the HFD + OVA and HFD + OVA + PLB group (Figure 2A; HFD + OVA group vs. OVA group and HFD + OVA + PLB group vs. OVA + PLB group: *p* <  .001). After 2‐week PLB treatment, the body weight of HFD‐fed mice (obese mice) significantly reduced (Figure 2B; HFD + OVA + PLB group vs. HFD + OVA group: *p*  <  .001). HFD feeding also increased epididymal fat mass, and the increases were inhibited by PLB treatment in obese asthmatic mice (Figure 2C; HFD + OVA group vs. OVA group: *p* <  .001; HFD + OVA + PLB group vs. HFD + OVA group: *p* <  .05). However, PLB treatment had no significant effect on the body weight and epididymal fat mass of nonobese asthmatic mice (Figure 2B and C; OVA + PLB group vs. OVA group: *p* >  .05). These findings indicated the weight‐reducing effect of PLB on obese mice.

### 3.2 PLB reduced peribronchial inflammation in obese and nonobese asthmatic mice

**Figure 3:**
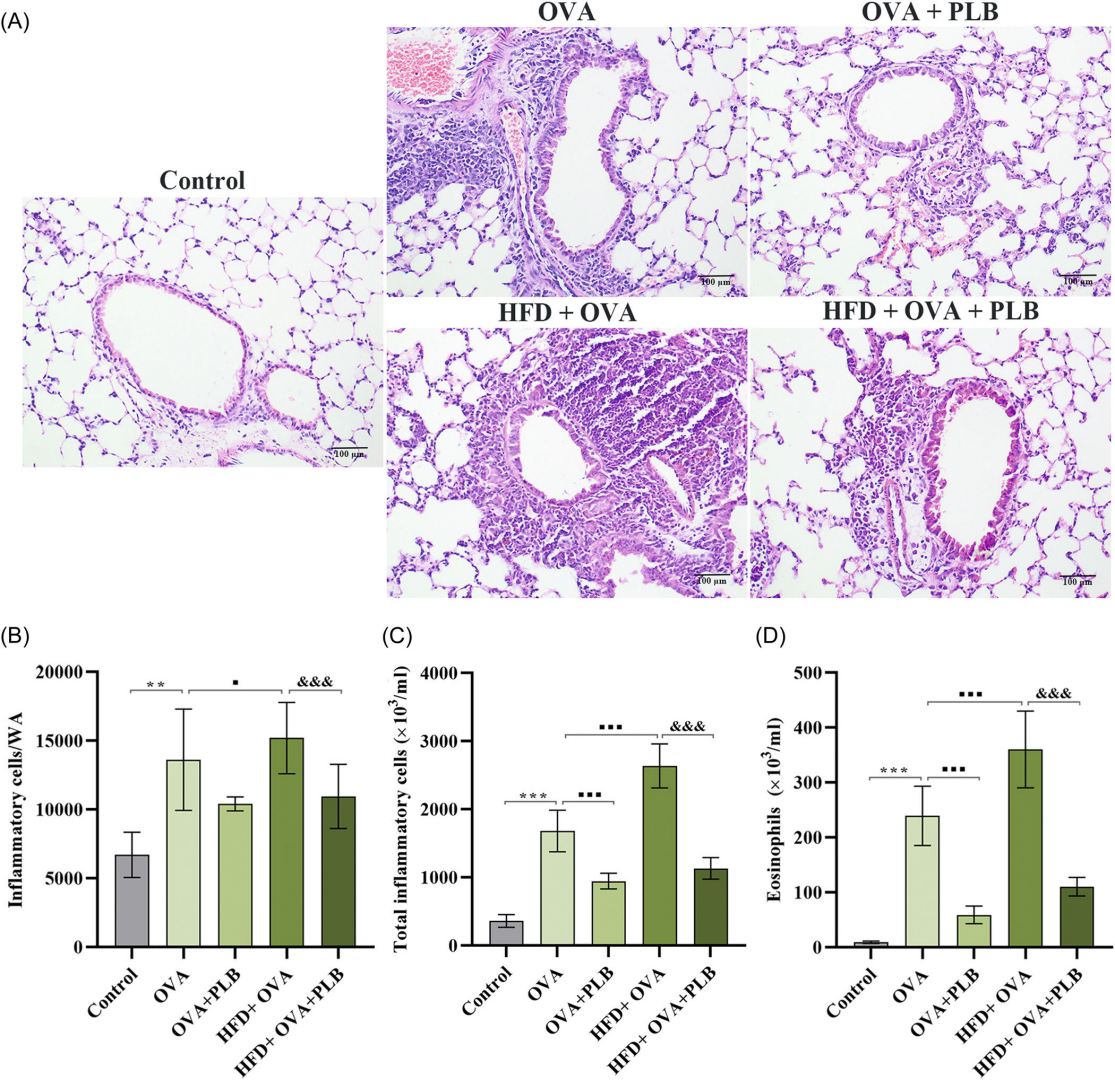
Effects of PLB on asthma‐related inflammation in nonobese and obese asthmatic mice. (A, B) The lung morphology was detected by H&E. Scale bar: 100 μm. Representative images were displayed. The number of inflammatory cells per wall area (WA, mm^2^) was quantified. (C) The number of total inflammatory cells and (D) eosinophils in BALF. **p < .01 and ***p < .001 versus Control group, ^■^ *p* < .05 and ^■■■^ *p* < .001 versus OVA group, ^&&&^ *p* < 0.001 versus HFD + OVA group. Data were mean ± SD. *N* = 6. BALF, bronchoalveolar lavage fluid; HFD, high‐fat diet; OVA, ovalbumin; PLB, plumbagin.

To determine whether PLB reduced peribronchial inflammation, the lung morphology was assessed using H&E staining (Figure 3A), and the inflammatory cells within the airway WA were counted (Figure 3B). The airway wall in obese asthmatic mice exhibited a higher level of inflammatory cell infiltration than that in nonobese asthmatic mice (Figure 3B; HFD + OVA group vs. OVA group: *p* < .05). In addition, we assessed the total inflammatory cell and eosinophil counts in BALF. The total inflammatory cell number and the eosinophil number were elevated in BALF from asthmatic mice (Figure 3C,D; OVA group vs. control group: *p* < .001). The elevations were more significant in obese asthmatic mice than in nonobese asthmatic mice (Figure 3C,D; HFD + OVA group vs. OVA group: *p* <  .001). The results suggested that HFD might exacerbate asthma‐related inflammation. The inhibitory effect of PLB on inflammatory cell infiltration in the airway wall was remarkable in obese asthmatic mice (Figure 3A,B; HFD + OVA + PLB group vs. HFD + OVA group: *p* <  .001). Peribronchial inflammation in nonobese mice had decreasing trends after PLB treatment (Figure 3A,B; OVA + PLB group vs. OVA group: *p* >  .05). PLB treatment led to significant reductions in total inflammatory cell and eosinophil counts in BALF from obese and nonobese asthmatic mice (Figure 3C,D; OVA + PLB group vs. OVA group and HFD + OVA + PLB group vs. HFD + OVA group: *p*  < .001). These results suggested that PLB probably reduced the peribronchial inflammation associated with asthma.

### 3.3 PLB reduced the levels of inflammatory cytokines in obese asthmatic mice

**Figure 4:**
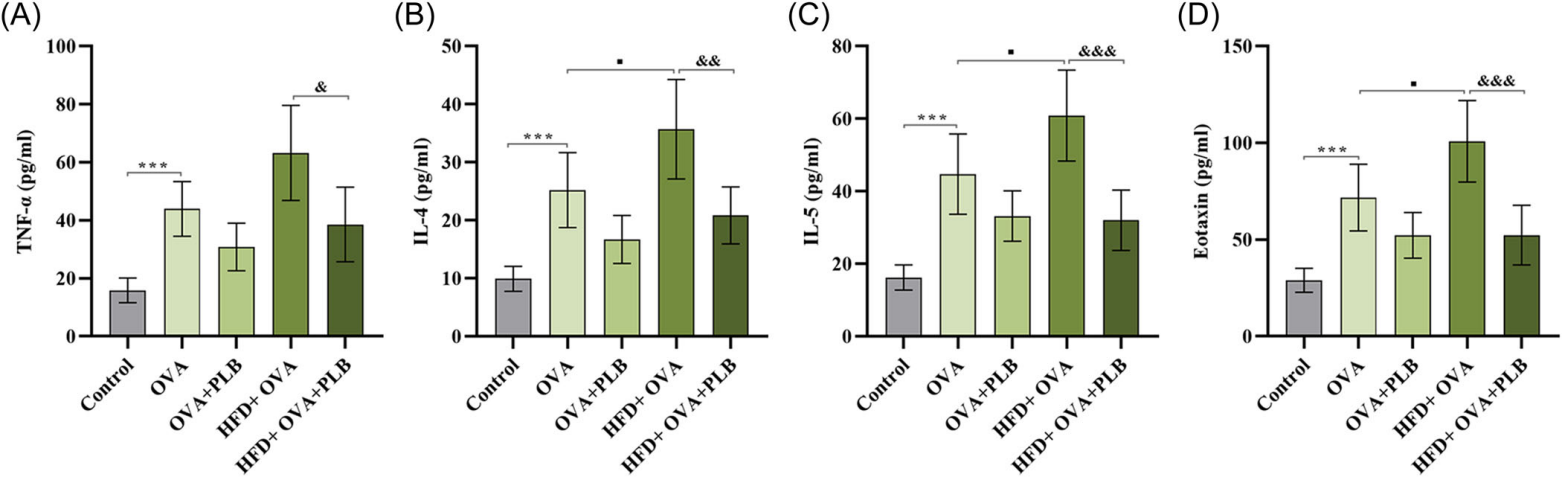
Effects of PLB on the levels of inflammation‐related cytokines in nonobese and obese asthmatic mice. (A) Tumor necrosis factor‐α (TNF‐α), (B) interleukin‐4 (IL‐4), (C) interleukin‐5 (IL‐5), and (D) eotaxin levels in BALF were evaluated by ELISA. ***p < .001 versus Control group, ^■^ *p* < .05 versus OVA group, ^&^ *p* < .05, ^&&^ *p* < .01, and ^&&&^ *p* < .001 versus HFD + OVA group. Results were expressed as mean ± SD. *N* = 6. BALF, bronchoalveolar lavage fluid; HFD, high‐fat diet; OVA, ovalbumin; PLB, plumbagin.

To further verify the mechanism of PLB on reducing inflammation, ELISA assays were performed to analyze the levels of inflammatory mediators in BALF. The levels of TNF‐α, IL‐4, IL‐5, and eotaxin were increased in BALF from nonobese asthmatic mice compared with those from the control mice (Figure 4A–D; OVA group vs. control group: *p* < .001). Obese asthmatic mice showed higher levels of IL‐4, IL‐5, and eotaxin in BALF (Figure 4B–D; HFD + OVA group vs. OVA group: *p* <  .05). An increasing trend was shown in the TNF‐α levels in BALF from obese asthmatic mice, compared with those in BALF from nonobese asthmatic mice (Figure 4A; HFD + OVA group vs. OVA group: *p* >  .05). These results indicated that obesity might aggravate airway inflammation in asthmatic mice. PLB treatment tended to decrease the level of TNF‐α, IL‐4, IL‐5, and eotaxin in BALF from nonobese asthmatic mice, although there was no significant difference between the OVA + PLB and OVA group (Figure 4A–D; *p* >  .05). Notably, the increased levels of inflammatory cytokines in BALF from obese asthmatic mice could be significantly reduced by PLB (Figure 4A–D; HFD + OVA + PLB group vs. HFD + OVA group: TNF‐α, *p* <  .05; IL‐4, *p* <  .01; IL‐5, *p* <  .001; eotaxin, *p* <  .001). These findings further confirmed the inhibitory effect of PLB on the peribronchial inflammation in obese asthmatic mice.

### 3.4 PLB alleviated oxidative stress in the lung tissues of obese and nonobese asthmatic mice

**Figure 5:**
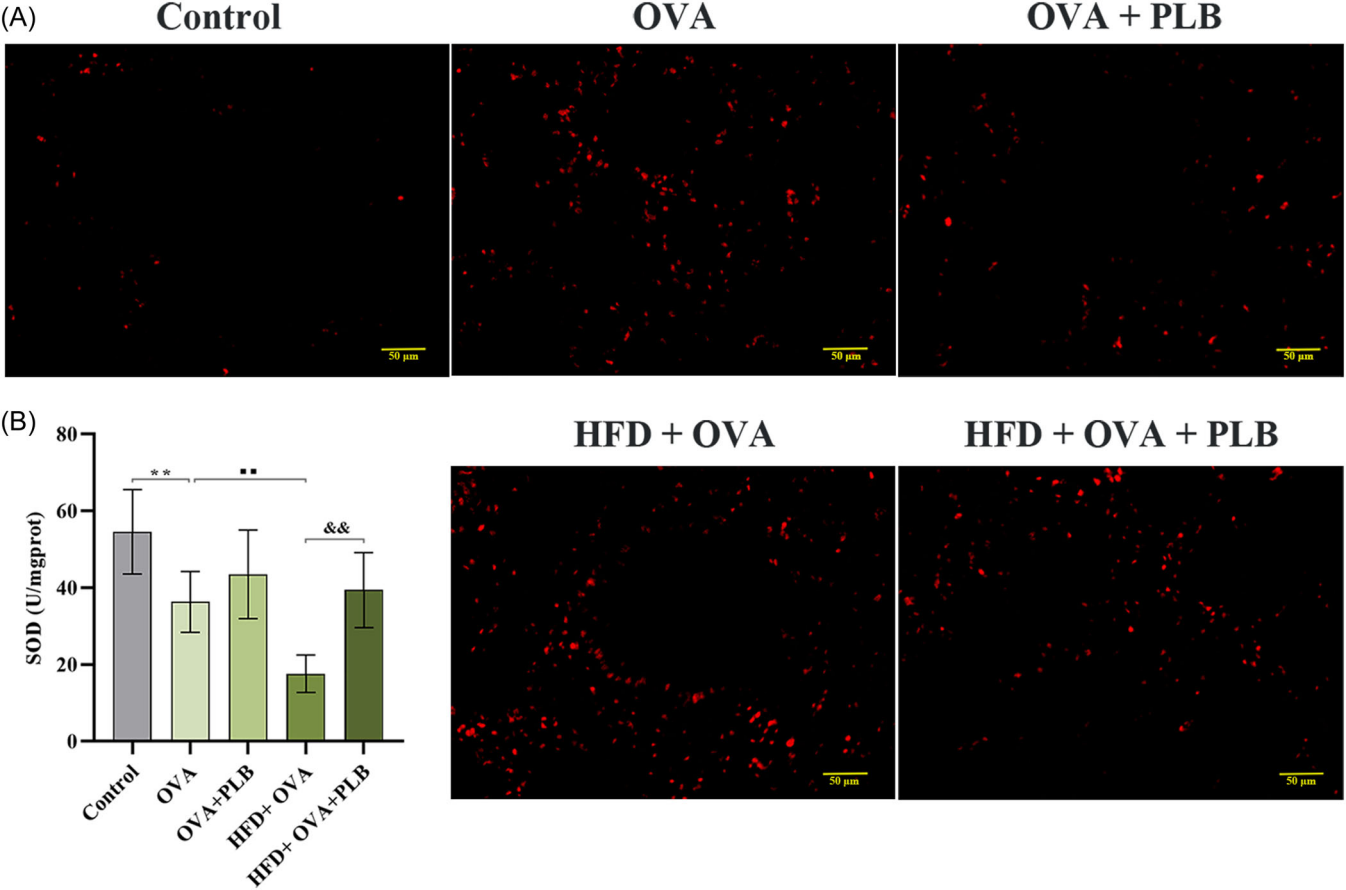
Effects of PLB on oxidative stress in the lung tissues of nonobese and obese asthmatic mice. (A) The BBoxiProbe® O13 probe was used to detect ROS levels. Representative images were acquired using a microscope. Scale bar: 50 μm. (B) SOD activities were measured by the SOD assay kit. **p < .01 versus Control group, ^■■^ *p* < .01 versus OVA group, ^&&^ *p* < .01 versus HFD + OVA group. Data were expressed as mean ± SD. *N* = 6. BALF, bronchoalveolar lavage fluid; HFD, high‐fat diet; OVA, ovalbumin; PLB, plumbagin; ROS, reactive oxygen species; SOD, superoxide dismutase.

ROS levels and SOD activities in the lung tissues were assessed to analyze the effect of PLB on oxidative stress in asthma and obesity‐related asthma. As results shown in Figure 5A, OVA challenge increased ROS accumulation in the lung tissues of obese and on‐obese asthmatic mice. PLB treatment suppressed the OVA‐induced accumulation of ROS. The activity of SOD in the lung tissues was measured. Pulmonary SOD activities were inhibited in OVA‐challenged mice (Figure 5B; OVA group vs. control group: *p* < .01). HFD further exacerbated OVA‐induced inhibitions of SOD activities (HFD + OVA group vs. OVA group: *p* <  .01), while PLB treatment partly restored the inhibitions of SOD activities in obese asthmatic mice (Figure 5B; HFD + OVA + PLB group vs. HFD + OVA group: *p* < .01). Therefore, PLB alleviated oxidative stress in asthma.

### 3.5 PLB activated the AMPK pathway in the lung tissues of obese and nonobese asthmatic mice

**Figure 6:**
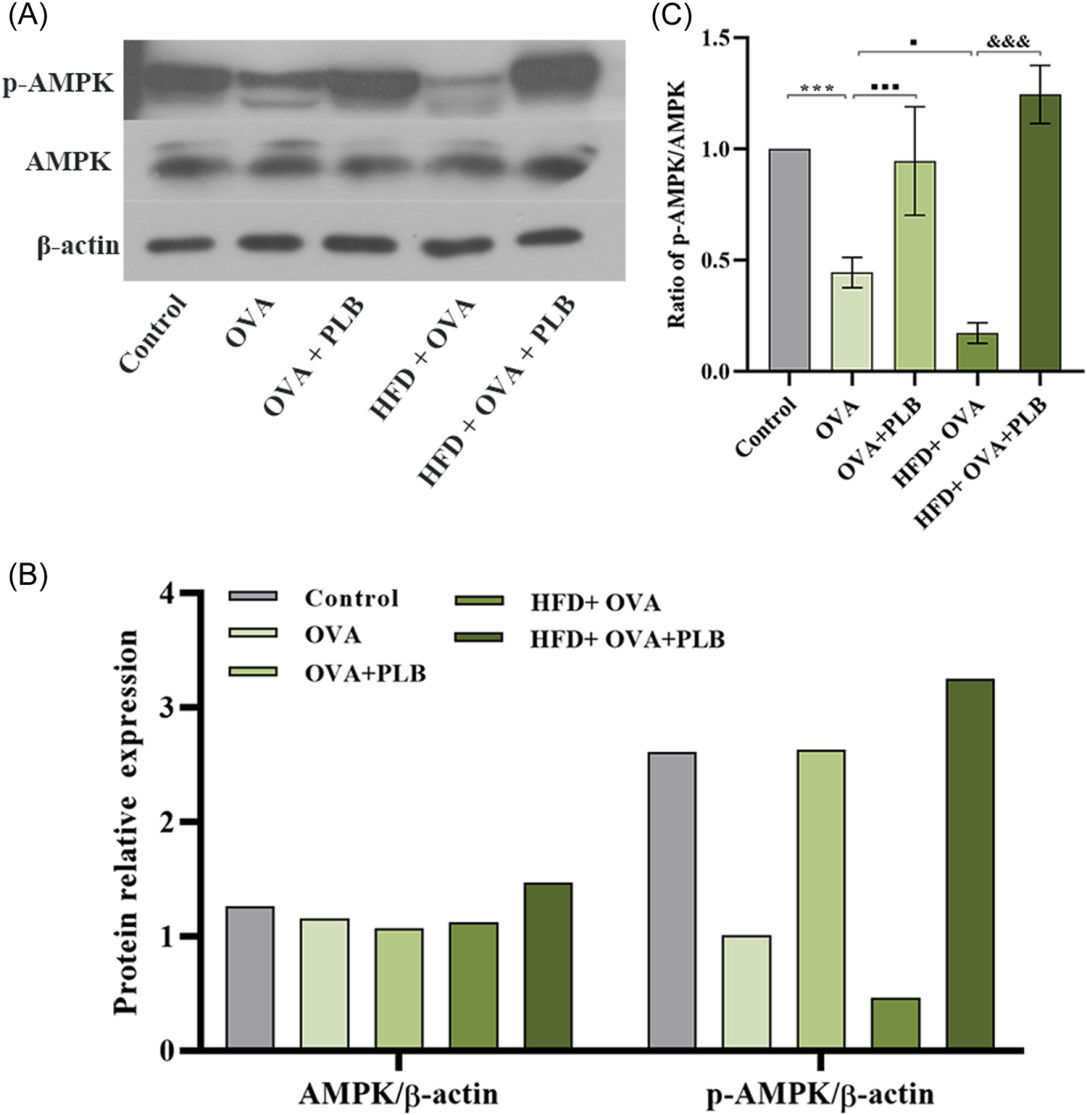
Effects of PLB on the AMPK pathway in the lung tissues of nonobese and obese asthmatic mice. (A) Representative immunoblots showed the protein expression of AMPK and p‐AMPK in the lung tissues. (B) The quantification of western blot. (C) The ratio of p‐AMPK/AMPK in the lung tissues. ***p < .001 versus Control group, ^■^ *p* < .05 and ^■■■^ *p* < .001 versus OVA group, ^&&&^ *p* < .001 versus HFD + OVA group. Data were expressed as mean ± SD. *N* = 6. AMPK, adenosine monophosphate‐activated protein kinase; HFD, high‐fat diet; OVA, ovalbumin; PLB, plumbagin.

The phosphorylation of AMPK in the lung tissues of asthmatic mice were reduced, and HFD aggravated the reductions in AMPK phosphorylation (Figure 6A–C; OVA group vs. control group: *p* < .001; HFD + OVA group vs. OVA group: *p* <  .05). PLB treatment increased AMPK phosphorylation levels in nonobese and obese asthmatic mice (Figure 6A–C; OVA + PLB group vs. OVA group and HFD + OVA + PLB group vs. HFD + OVA group: *p* < .001). These findings confirmed the activation of AMPK pathway by PLB in asthmatic mice.

## 4 DISCUSSION

Obesity‐related asthma is a special type of asthma. The Global Initiative for Asthma (GINA) proposed that obesity‐related asthma was considered a new disease in 2014.^27^ Compared with other types of asthma, obesity‐related asthma has more severe clinical manifestations and a poor response to asthma medication such as glucocorticoids. Obesity‐related systemic inflammation and oxidative stress are closely associated with asthma exacerbations in obese patients.^9^, ^28^ Pai et al.^29^ found that 8‐week PLB treatment could reduce obesity. Our study showed that obese asthmatic mice lose their weight after 2‐week PLB treatment, indicating short‐term PLB treatment also could alleviate obesity. Asthma is a chronic airway inflammation characterized by leukocyte infiltration and shedding of airway mucosal surface epithelial cells.^30^, ^31^ Asthmatic mice exhibited inflammatory symptoms due to the infiltration of inflammatory cells into the airway, particularly eosinophil infiltration.^32^ Our study found that eosinophil counts in BALF and inflammatory cell infiltration in the airway walls of asthmatic mice were more apparent in obese asthmatic mice than in nonobese asthmatic mice. Moreover, obese asthmatic mice exhibited greater inflammatory responses. IL‐4, IL‐5, and eotaxin are related to eosinophil inflammation.^33^ In asthma, T helper type 2 (Th2) cells, basophils, or mast cells were a source of IL‐4 and IL‐5. Anti‐IL‐4 and anti‐IL‐5 treatment attenuated the infiltration of eosinophils in the airways of OVA‐challenged mice.^34^ Cytokines, such as IL‐4, stimulate smooth muscle cells, epithelial cells, and fibroblasts to secret eotaxin, thereby increasing the recruitment of eosinophils.^35^ Therefore, obesity‐induced increases in eosinophil inflammation in the airways of asthmatic mice may be linked to the increased production of IL‐4, IL‐5, and eotaxin. TNF‐α plays an important role in asthma and the inhibition of TNF‐α is beneficial for the treatment of chronic inflammation.^35^ We found an increasing tendency in TNF‐α levels in obese asthmatic mice, indicating that HFD also might affect eosinophil inflammation partly by increasing TNF‐α levels. In addition, it is clear that obesity‐induced systemic oxidative stress is associated with poor control of asthma in obese asthmatics.^28^ ROS directly regulates airway smooth muscle contraction and further generates AHR.^36^ SOD is an antioxidant enzyme that can be inactivated by ROS.^37^ SOD inactivation exacerbates inflammation and airway obstruction, which is related to the elevated levels of oxidative stress in asthmatics.^17^ Herein, the activity of SOD was suppressed, while the ROS levels were increased in the lung tissues of nonobese and obese asthmatic mice. Interestingly, more significant decreases in SOD activities and increases in ROS levels were observed in obese asthmatic mice, which indicated that obesity probably exacerbated oxidative stress in asthmatic mice. Consistently, Liang's findings^38^ also suggested that obesity caused pathophysiological changes and exacerbated airway inflammation.

There is a controversy surrounding obesity and asthma. Sideleva et al.^12^ considered that obesity might contribute to asthma by directly affecting the airways rather than enhancing inflammation to indirectly affect the airways. Obesity‐related asthma presumably is divided into two categories: the early‐onset disease complicated by obesity and the late‐onset asthma that may develop due to obesity. The relationship between obesity and asthma remains complex and unclear. The divergence of basic research drove us to further explore their relationship. It has been proved that the inhibition of proinflammatory mediator production could alleviate the development of allergic asthma.^39^ Our findings showed that PLB treatment decreased inflammatory cell counts in BALF and led to the reduction in the circulating levels of eosinophil‐related inflammatory mediators (IL‐4, IL‐5, and eotaxin) and TNF‐α in obese asthmatic mice. We speculated that PLB might affect inflammatory cell accumulation in obese asthmatic mice by regulating these medaitors. In nonobese asthmatic mice, PLB treatment significantly decreased inflammatory cell counts in BALF, while PLB had no significant effect on circulating levels of IL‐4, IL‐5, and eotaxin, indicating that PLB might affect inflammatory cell accumulation not just by regulating these medaitors. In addition to IL‐4 and IL‐5, granulocyte‐macrophage colony‐stimulating factor (GM‐CSF) is also an important mediator driving eosinophil accumulation in airway inflammation in asthma.^40^ Emerging evidence has suggested that PLB could inhibit GM‐CSF expression.^41^ However, it remains unclear whether PLB can affect inflammatory cell accumulation in nonobese asthmatic mice by regulating other mediators such as GM‐CSF, which is a limitation for the present study and needs to be explored in the subsequent work. In addition, PLB alleviated the imbalance of oxidative stress in obese asthmatic mice. Previous experimental studies showed that PLB could not only inhibit oxidative stress^21^, ^29^, ^42^ but also reduced IgE‐induced allergic responses.^22^ The above results suggested that PLB probably alleviated obesity‐related asthma by suppressing inflammation and oxidative stress.

AMPK, a metabolism‐sensitive protein kinase, is a key energy sensor regulating cellular metabolism and it is important for obesity.^43^ Activated AMPK inhibited the inflammatory process.^44^ In this work, we observed that the phosphorylation of AMPK was inhibited in the lung tissues derived from obese asthmatic mice, and the inhibition was reversed by PLB treatment. It was consistent with Wang's research.^23^ Recent studies reported that activated AMPK enhanced the activity of antioxidant enzymes, attenuated oxidative stress, and thereby exerted protective effects by inhibiting ROS production.^45^, ^46^ Our results showed that PLB promoted AMPK activation in obese asthmatic mice, which implied that PLB might alleviate oxidative stress by activating the AMPK pathway in obesity‐related asthma. Zhu et al.^24^ also found that activated AMPK inhibited oxidative stress and further alleviated allergic asthma. These findings suggested that the effects of PLB during anti‐inflammatory and antioxidant processes depended, at least in part, on AMPK activation in obesity‐related asthma.

In brief, the results revealed that obesity possibly exacerbated inflammation and oxidative stress and PLB treatment relieved obesity‐related asthma by suppressing oxidative stress, ameliorating inflammation, and activating the AMPK pathway. Thus, PLB may serve as a potential drug candidate for treating obesity‐related asthma.

## AUTHOR CONTRIBUTIONS

**Lijie Zhang**: Conceptualization; visualization; writing—original draft. **Dongxue Liang**: Conceptualization; formal analysis; investigation; validation. **Linlin Liu**: Conceptualization; formal analysis; investigation; validation. **Lihua Liu**: Conceptualization; funding acquisition; supervision; writing—review & editing.

## CONFLICT OF INTEREST STATEMENT

The authors declare no conflict of interest.

## Supporting information

Supporting information.

## Data Availability

The data used in this study are available from the corresponding author upon reasonable request.
