# Supplementary material for: Plumbagin alleviates obesity‐related asthma: Targeting inflammation, oxidative stress, and the AMPK pathway
Source: Immun Inflamm Dis. 2023 Sep 27;11(9):e1025. doi: 10.1002/iid3.1025 (PMC10524032; doi:10.1002/iid3.1025)
Supplement: Supplementary file 1 — Supporting information. [file IID3-11-e1025-s001.docx]

**Supplementary materials**

Table S1 The number of mice used for experimental analysis.

|  | Control | OVA | OVA+PLB | HFD+OVA | HFD+OVA+PLB |
| --- | --- | --- | --- | --- | --- |
| Total | 12 | 17 | 17 | 17 | 17 |
| Mice without asthma-like symptoms | N/A | 4 | 5 | 5 | 4 |
| Unexplained death | None | 1 | None | None | 1 |
| Mice used for experimental analysis | 12 | 12 | 12 | 12 | 12 |

N/A: not applicable.
